# The complete chloroplast genome of an evergreen species *Camellia japonica*

**DOI:** 10.1080/23802359.2019.1627926

**Published:** 2019-07-11

**Authors:** Wei Li, Cui-Ping Zhang, Kui-Ling Wang

**Affiliations:** College of Landscape Architecture and Forestry, Qingdao Agricultural University, Qingdao, China

**Keywords:** *Camellia japonica*, chloroplast genome, phylogenetic relationships

## Abstract

*Camellia japonica* is an evergreen tree species with high ornamental value. The complete *C. japonica* cp genome is 156,606 bp in length and contains a small single-copy region (18,415 bp) and a large single copy ( 86,257 bp) region – separated by a pair of the inverted repeat regions (51,934 bp ). The overall GC content of the *C. japonica* cp genome is 37.31%. We identified 128 genes in this genome, including 91 protein-coding genes, 29 transfer RNA genes, and 8 ribosomal RNA genes. The maximum-likelihood phylogenetic analysis revealed that *C. japonica* is closely related to *Camellia oleifera*.

*Camellia japonica* L. is the only *Camellia* species found in the temperate zone in China. It is well-known for its ornamental value and has been widely used for horticultural gardening. However, wild *C. japonica* have been seriously harmed by human activities and developing strategies for their conservation is an urgent task. DNA markers have previously been used for the phylogenetic analyses, genetic diversity evaluation, and plant molecular identification of *Camellia* (Intrieri et al. 2007; Hwang and Yong 2016; Sarra, et al. 2017). In this study, we aim to retrieve valuable genomic information of the complete *C. japonica* cp genome through next-generation sequencing (Du et al. 2015; Lu et al. 2018).

Fresh leaf tissues were collected from the Daguan Island in Qingdao of Shandong province, China (36°14′ N, 120°46′ E). The voucher specimen was deposited in Qingdao Agricultural University. Total genomic DNA was extracted according to the method of Li and colleagues (Li et al. 2013). A DNA sample was randomly fragmented to construct paired-end (PE) libraries according to the Illumina preparation manual (Illumina, San Diego, CA).

Approximately 1.7 Gb raw data were generated with paired-end 150 bp reads sequencing. We assembled the cp genome using CLC Genomics Workbench v7.5 (CLC Bio, Aarhus, Denmark). The assembled cp genome was then annotated by DOGMA (Zhang et al. 2019). The complete *C. japonica* cp genome sequence has been submitted to GenBank with the accession number PRJNA510919.

The complete *C. japonica* cp genome is 156,606 bp in length. This genome contains a large single copy region of 86,257 bp and a small single copy region of 18,415 bp. The LSC and SSC regions are separated by two inverted repeat regions – each is 51,934 bp in length. The overall GC content of the complete cp genome is 37.31% and local GC contents for the LSC, SSC, and IR regions are 35.34, 30.53, and 43.01%, respectively. The entire cp genome encodes 128 genes, including 91 protein-coding genes, 29 tRNA genes, and 8 rRNA genes. Among the 128 genes, 10 genes contain only one intron and 2 genes were identified with two introns. Gene duplication was observed for seven protein-coding genes, eight tRNA genes, and four rRNA genes in the IR regions and the rest of the genes have only a single copy.

Due to the controversy over the interspecific relationship of the genus *Camellia* (Vijayan et al. 2009; Yang et al. 2013), we reconstructed the phylogenetic tree of the *Camellia* species. A total of 19 cp genomes were subjected to the phylogenetic analysis – the *C. japonica* cp genome obtained in this study and 18 other *Camellia* cp genomes obtained from the NCBI. This analysis indicates that *C. japonica* is most closely related to *Camellia oleifera* (Figure. 1). Furthermore, the phylogenetic analysis is consistent with the section-level classification by Min and Bartholomew (2007). Our study provides valuable genetic information of the *C. japonica* cp genome and clarifies the phylogenetic relationship between *C. japonica* and other *Camellia* species. Findings of this research will inform the conservation, taxonomy, and breeding approaches of *C. japonica* and the genus *Camellia*.

**Figure 1.**
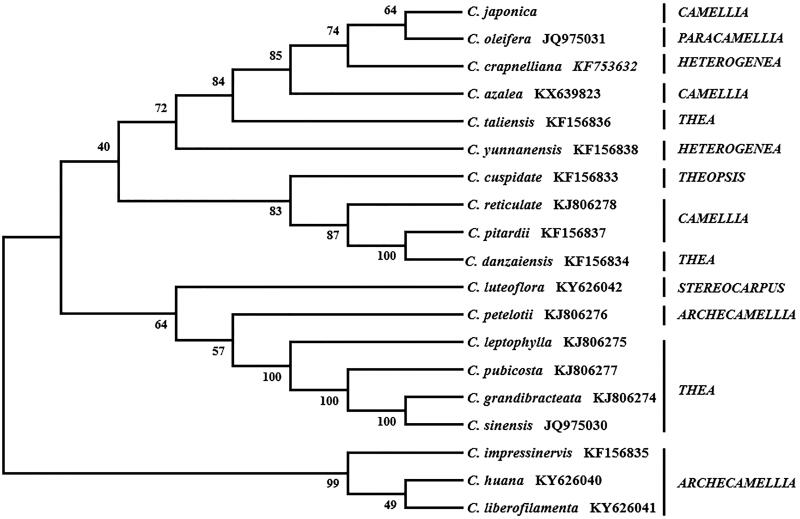
Phylogenetic relationships of the 19 *Camellia* species constructed from the complete chloroplast genome sequences.
